# Review: On Segmentation of Nodules from Posterior and Anterior Chest Radiographs

**DOI:** 10.1155/2018/9752638

**Published:** 2018-10-18

**Authors:** S. K. Chaya Devi, T. Satya Savithri

**Affiliations:** ^1^JNTU College of Engineering, Hyderabad, India; ^2^Department of E.C.E, JNTU College of Engineering, Hyderabad, India

## Abstract

Lung cancer is one of the major types of cancer in the world. Survival rate can be increased if the disease can be identified early. Posterior and anterior chest radiography and computerized tomography scans are the most used diagnosis techniques for detecting tumor from lungs. Posterior and anterior chest radiography requires less radiation dose and is available in most of the diagnostic centers and it costs less compared to the remaining diagnosis techniques. So PA chest radiography became the most commonly used technique for lung cancer detection. Because of superimposed anatomical structures present in the image, sometimes radiologists cannot find abnormalities from the image. To help radiologists in diagnosing tumor from PA chest radiographic images range of CAD scheme has been developed for the past three decades. These computerized tools may be used by radiologists as a second opinion in detecting tumor. Literature survey on detecting tumors from chest graphs is presented in this paper.

## 1. Introduction

Lung cancer is one of the major types of cancer in both men and women. Prostate cancer in men and breast cancer in women are the most common. According to American cancer society reports, about 14% of new cancers are lung cancers and their estimations in United States during 2018 were about 234,030 and about 154,050 deaths from lung cancer (83,550 in men and 70,500 in women). Recent study from World Health Organization shows that there are about 7.6 million deaths every year due to lung cancer, and mortality rate may continuously increase, becoming around 17 million approximately worldwide by 2030.

Early detection increases the chances of survival. Posterior and anterior chest radiography and computerized tomography techniques are used for detecting tumor from lungs. Out of all these techniques, frequently used diagnosis technique is PA chest radiography because it requires less radiation dose, costs less, and is mostly available in almost every diagnostic center. Due to complex anatomical structure present in the image radiologists find difficulty in diagnosing tumor from PA chest radiographic images. To help radiologists in detecting tumor from chest radiographs, Computer Aided Diagnosis systems have been developed for decades. These tools can be used by radiologists as second opinion in detecting tumor. CAD, if it works well, speeds up the diagnostic process and advances the qualitative valuation.

Objective of this paper is to briefly review the literature on computer analysis of posterior and anterior chest radiographs. Research is done on two main areas:

1. ***Preprocessing &. Lung segmentation***
2. ***Nodule detection***
  - Candidate detection
  - Feature extraction & classification

This paper is organized as follows: in Section 1 introduction about lung cancer and diagnosis technique were discussed, in Section 2 preprocessing and lung segmentation followed in different methods are described, Section 3 consists of various nodule detection techniques, and paper ends with conclusions in Section 4.

## 2. Preprocessing and Lung Segmentation

### 2.1. Preprocessing

**Figure 1:**
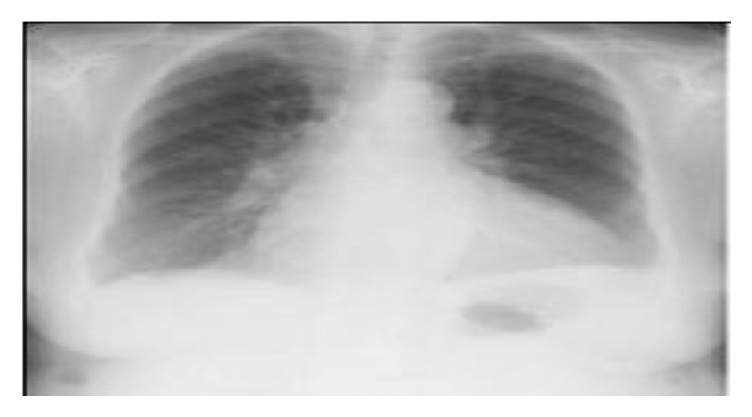
Original image.

**Figure 2:**
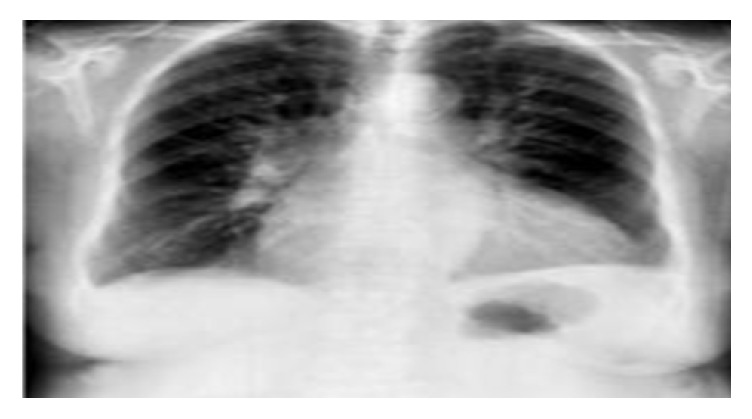
Enhanced image.

Computerized analysis of P.A chest radiographs begins with preprocessing the image. Scanned images are resized and resampled to a fixed resolution and noise is removed if required. The purpose of the preprocessing is to make the image suitable for further processing. Enhancement done with different methods like local contrast enhancement, global contrast enhancement [1], and also homomorphic filtering [2] enhanced image shown in Figure 2. The original image is shown in Figure 1.

### 2.2. Lung Segmentation

**Figure 3:**
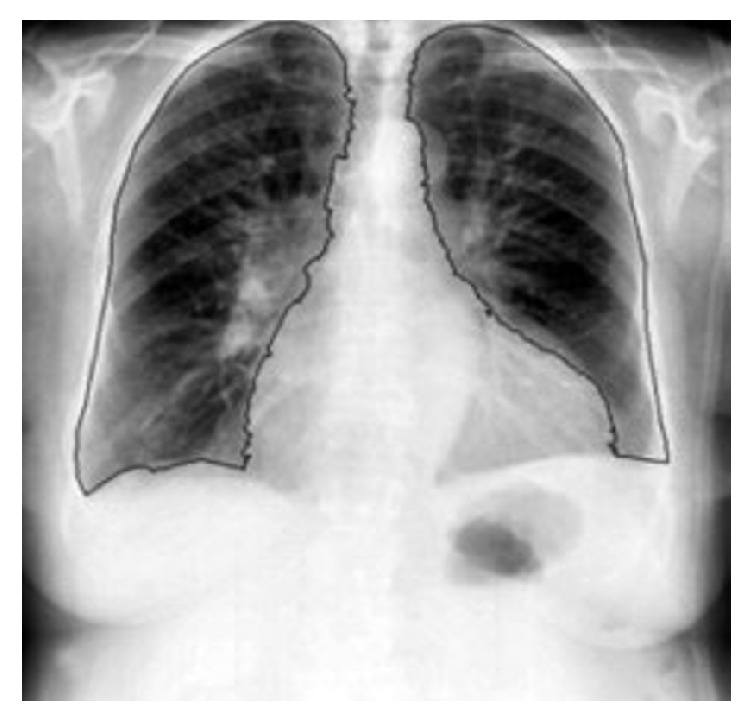
Segmented image.

After preprocessing image, visible lung region is segmented from the image. This is done to reduce the processing area of the image. However incomplete segmentation may lead the CAD system to overlook lesion in unsegmented area. Segmentation done mainly using rule based methods, pixel classification, and knowledge based methods. Segmented image using active shape modeling is shown in Figure 3.

Rule based scheme follows certain processing steps using some adjustable parameters; these methods were presented by S. G. Armato, M. L. Giger, and H. MacMahon* et.al* [3] X. W. Xu and K. Doi* et.al* [4] F. M. Carrascal, J. M. Carreira, M. Souto, P. G. Tahoces, L. Gomez, and J. J. Vidal* et.al* [5] E. Pietka,[6]M S. Brown, and L. S. Wilson* et.al* [7].

S.G.Armato [3] and coworkers used a range of threshold values obtained from gray level histogram analysis for performing iterative global grey level thresholding technique. At the output of grey level thresholding, local thresholding was applied. Resulted contours were smoothed with several smoothing techniques including rolling ball technique. Method was tested on 600 PA chest radiographs. Segmentation accuracy published was 79%. X. W. Xu and K. Doi [4] followed different rule based segmentation technique, where initially average position of the top of the lung is found and then within ROI top lung edges and rib cage edges were determined. Three polynomial functions were applied on image independently to obtain smooth curves for top lung edges and rib edges. Reportedly technique was tested on 1000 images with 96% accuracy. The same authors in their continued study presented another technique [8] in which left and right diaphragm edges together with ribcage edges were found using edge gradient analysis and with some standard rules. Polynomial functions were applied to smooth the curves. Finally segmentation of the lung fields was acquired by joining the left and right hemi diaphragm edges curves with the equivalent rib cage edges curves. According to published results accuracy obtained was approximately 97% and 90%. F. M. Carrascal and others [5] presented another rule based method for segmenting lung fields, in which, using a group of reference lines, a family of ROIs are obtained, which consists of pulmonary borders. In each ROI pulmonary borders were recognized using edge enhancement and thresholding methods. Pulmonary borders were corrected and completed with the help of interpolation, extrapolation, and arc fitting techniques. Comparing these automatic lung tracing results with the manual lung tracing, expert radiologists precision obtained was 0.990±0.001 E. Pietka [6] proposed one more structural technique for lung segmentation, in which background anatomical structure was removed by employing histogram thresholding and gradient analysis. Smooth lung edges were obtained after applying cubic spline interpolation procedure and morphological erosion operation. Final segmented lungs were obtained after applying dilatation operation. Technique tested on 40 images belongs to adults and children, and results show correct segmentation obtained for 35 images. M. S. Brown and L. S. Wilson* et.al* [7] proposed a knowledge method for segmentation where low level object was compared with the high level objects using intermediate representation called parametric features. The high level objects are described as anatomical model. Both normal and abnormal feature values are used for modeling so that method can interpret the abnormal variations effectively. The system showed 88% sensitivity with specificity of 95%, when compared with an evaluation by radiologists.

Lung segmentation with pixel classification by employing neural networks was proposed by McNitt-Gray* et. al *[9], Osamu Tsujii, MS Matthew* et. al *[10] N. F. Vittitoe, R. Vargas-Voracek, and C. E. Floyd et. al [11], Bram van Ginnekena, and Bart M. ter Haar Romeny [12].

McNitt-Gray* et. al *presented segmentation method that employs pattern classification approach to segment anatomic regions like lungs and heart. Three types of classifier performances were compared, 17 chest images used for training, and 16 different chest images used for testing. Linear discriminant classifier and k-nearest neighbor classifier can distinguish patterns with an efficiency of more than 70% and neural network with efficiency greater than 76%. The same authors [13] in their next proposed technique used locally calculated features to classify pixels into one of the several anatomical classes. Since subset of features that were used for classification gives reduced computational complexity as well as reduced time with efficiency comparable to the full set of features, Osamu Tsujii et.al [10] proposed a method for automatic lung segmentation; initial size of the image is reduced and smoothed; in the next step, image is resolved into horizontal and vertical profiles. These profiles were given as input for two convolution neural networks. Networks were trained using vertical profiles and horizontal profiles of 14 images including images used for testing. Output profiles of each neural network were reconstructed into two dimensional images. After binarization, the two images are combined with OR operation. Techniques efficiency was reported as 94%. N. F. Vittitoe, R. Vargas-Voracek, and C. E. Floyd [11] developed a method using Markov random field modeling, where Markov random field model is developed by utilizing spatial and textural information extracted from samples of lung and nonlung region. With the help of this model classification of pixels in each image is done. The algorithm works with sensitivity of 90.7%, a specificity of 97.2%, and an accuracy of 94.8%. Bram van Ginnekena and Bart M. ter Haar Romeny [12] presented hybrid method which combines both rule based and pixel classification approaches. Accuracy of the proposed scheme is .969 for all 115 images of the test set.

Visible lung region can also be segmented from PA chest radiographic images using different knowledge based techniques like active shape modeling, active contour modeling, and active appearance modeling. Active shape models are statistical shape models, also called deformable models which can build by analyzing variations in shape over a set of images. This can allow us to study new shapes and to create shapes similar to those in the training set. Training set is built by hand annotation of set of images. Initial active shape was model developed by T.F.cootes. Further developments and applications on ASM were done by the remaining authors. T.F.cootes et.al [14] modified their original algorithm, which can adapt to the shape variability of the object to be detected, but is specific to the particular class of structures technique which uses Mahalanobis distance, to find optimal displacements for landmarks. Bram van Ginneken et.al [15, 16] used nonlinear KNN classifier for finding optimal displacement for land mark points. Technique adapts automatic feature selection. Method was applied on 234 chest radiographs for left and right lung field segmentation, which are available in database. Deformable model developed by Yonghong Shi et.al [17] makes use of the features around the lung boundaries extracted using scale invariant feature transform [SIFT] local descriptor for segment the lung fields. The authors state that the algorithm is more efficient compared to ASM. Yonghong Shi et.al [18] extended their work [17] and proposed a different deformable model, which uses population based and patient specific shape statistics. Image features at neighborhood of each pixel are characterized by scale invariant feature transform (SIFT) local descriptor unlike general intensity and gradient features. Deformable contours are forced to learn the population based shape statistics and also with patient specific shape statistics as segmentation proceeds. The authors declared that the algorithm is more robust and accurate than other active shape models.

The most demanding issue in applying active contour models for segmentation of lung area from PA chest radiographs is local minima; this is because strong edges belong to rib cage and clavicles and due to shading effects. P.Annangi, S.Thiruvenkadam, and A.Raja* et.al *[19] used contrast of the lung boundaries to derive multiscale set of edge/corner feature points and active contour models are driven with these features. The authors found local minima issues solved by adding these features with region based data and average lung shape. Algorithm tested on 1130 clinical cases shows efficiency of 88% in comparison with manual segmentation. Seghers* et.al *[20] presented a method in which shape and intensity characteristics are optimized parallelly throughout the search; this technique is different from ASM. A landmark identifier was defined; it assigns a value to the point in the image, which represents the relation between gray level appearances at the landmark point and the probable intensity pattern of the landmark as derived from training images. These values were stored as intensity cost. Landmarks with low intensity cost are considered as landmark points of interest. Both shape and intensity cost functions were combined and optimized using dynamic programming. Method was tested on 247 JSRT images.

Proposed methods were tested on different databases so segmentation results of one method cannot be compared with the other. No segmentation algorithm in the literature gives 100% results. Segmentation methods proposed by Bram van Ginnekena et.al [12, 15, 16] show segmentation accuracy of 0.969, which may be the best segmentation technique.

## 3. Nodule Detection

After delineating visible lung region from posterior and anterior chest radiographs, the next step is detecting potential nodule from the segmented image. Most of the authors followed three step processes for detecting nodule region, namely, suspicious nodule candidate detection, feature selection, and classification. The following sections describe these techniques.

### 3.1. Candidate Detection

**Figure 4:**
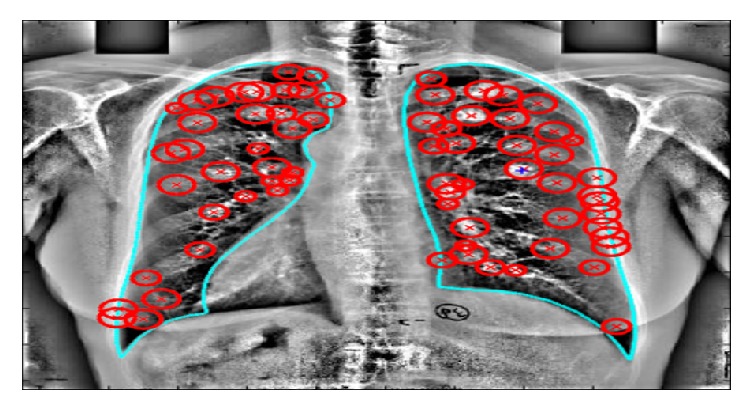
Suspected nodule regions.

These methods were proposed for finding suspected nodules. Detected nodule candidates may have disseminated abnormalities. This detection process consists of, for example, difference imaging technique and thresholding; some authors suspect possible nodule regions from the image by applying different filters like Laplacian of Gaussian and Gabor, ARG filter and Gaussian filter, Iris filter, and snake algorithm; a few authors used matching technique for finding possible regions. In some techniques nodule regions are selected with the help of radiologists. A few methods for finding suspected nodules from image were discussed here.  Figure 4 shows the suspected nodules detected from PA chest radiographic images.

Giger ML, Doi K, MacMohan H.*et.al *[21, 22]Wu Y, Doi K, and Giger ML,* et.al. *[23] employed difference imaging technique for removing background anatomic structures present in PA chest radiograph. From single chest image two filtered images were generated; in one filtered image nodule signals are enhanced and in another image nodule signals are suppressed. Difference of these images results in an image with nodule enhanced and background suppressed. And nodule regions were suspected, based on circularity and size of nodule after applying thresholding on difference image. Lo SC, Lou Sl, Lin JS,* et al*. [24] presented a CAD algorithm in which initial nodule candidate search was performed with sphere template double matching technique. Penedo M.G, Carreira M.J, Mosquera A,* et al.* [25] normalized the input image to enhance the nodule like structure in the image and multilayer perceptron with two hidden layers used to find suspicious regions. Network not only trained with real images with different nodule sizes, but also trained with simulated images having different nodule sizes. For each suspicious nodule regions detected, curvature peak features were manipulated. These are used for potential nodule candidate detection. Coppini G, Diciotti S, Falchini M,* et.al* [26], and [2, 27]used biologically inspired filters like Laplacian of Gaussian and Gabor filters to locate possible nodular regions. Zhenghao Shi, Minghua Zhao, Lifeng He, Yinghui Wang, and Ming Zhangand Kenji Suzuki* et.al* [28] considered shape of the nodule as spherical. For finding nodules of different sizes, Gaussian filter at different scales is applied, starting with small scale. Scale is gradually increased, and for each scale after applying Gaussian, eigenvalues are calculated, and with the help of eigenvalues suspected nodule candidates are determined. By using Rule based classifier with features like sphericity and effective diameter small nodule and elongated suspected nodule region were removed. Al Gindi A., Rashed E., Sami M.*et.al*, [29] take the help of 3 radiologists for ROI selection. ROIs (Region of Interest, i.e., nodule regions) extracted are of three different sizes, 128*∗*128, 64*∗*64, and 32*∗*32. Reason for selecting 3 different sizes is that nodules in the database were not of the same size. Chen S., K. Suzuki and H. McMahon* et.al*,[30] applied clustering watershed segmentation for detecting suspicious regions. Hardie R. C., S. K. Rogers, T. Wilson and A. Rogers,* et.al* [31] performed initially local contrast enhancement on each image for normalizing the contrast across different images and within the image. Weighted convergence index filter was applied on enhanced image to find suspected nodule candidates and adaptive distance based threshold is used to segment the nodule candidates. Shiraishi, Q. Li, K. Suzuki, R. Engelmann, and K. Doi,* et.al.*[32] segmented the lung region into 7*∗*7 sections, each section of fixed size 64*∗*64. Each ROI was classified into different anatomical regions based on location in the lung field like apical, peripheral, helium, and opaque areas. Nodule candidates were identified by considering search regions of size 128*∗*128. Nodule regions were enhanced using ARG filter and Gaussian filter. A. Schilham, B. van Ginneken, and M. Loog,* et.al*.[33, 34] applied Blob detection method on normalized and enhanced image for finding suspicious nodule regions. J. Wei, Y.Hagihara, A.Shimizu, and H.Kobatake.*et.al *[35] employed adaptive ring filter of type convergence index filter for identifying locations. Selected region boundary was identified in two-step process, Iris filter was employed to estimate fuzzy boundary, and then a snake algorithm was used on the output of the Iris filter to obtain the boundary of the nodule region. S.A.PATIL, M. B. Kuchanur* et.al*.[36, 37] presented an algorithm in which they used thresholding and region growing methods for finding suspicious regions in case of NSLC type cancers and for SCLC type cancers region labeling is employed. Zakaria Suliman Zubi, Rema Asheibani Saad* et al. *[38] applied thresholding and series of morphological operations on segmented visible lung region for finding suspected nodule candidates. Orbán,Á. Horváth and G. Horváth* et.al *[39] applied nodule detection method on rib suppressed images. Constrained sliding band filter was used to enhance the nodule regions. Regions which are having high CSBF value are considered as suspected nodule regions. K.A.G. Udeshani, R.G.N. Meegama and T.G.I. Fernando* et.al.*[40] applied otsu method on an enhanced image, which converts the image into binary image and circular index of each connected component was found to determine the possible nodule regions. Nitin S. Lingayat and Manoj R. Tarambal* et.al *[41] suspected the possible nodule regions using different image processing techniques like thresholding, edge detection, and labeling. Kim Le* et.al*.[42] published a different technique for finding suspected nodule candidates, in which for every lung pixel fixed size window is applied and average and maximum gray level values of the pixels inside the window were calculated. The value between average and maximum gray level values is selected as threshold value. Pixels which are having gray level values greater than threshold were marked. Number of pixels greater than threshold is counted; if the count is greater than predefined threshold that set of pixels is considered as suspected nodule. X. W. Xu* et al.*[43] employed a difference imaging and multiple gray level thresholding technique for finding possible nodule candidates.

### 3.2. Feature Extraction and Classification

In this stage each detected candidate is tested in more detail. And potential nodule candidate was extracted and false positive findings were reduced with basically two methods, i.e., features extraction and classification.

### 3.3. Feature Extraction

Each suspected nodule region is examined and potential candidate selected. Candidate selection is done by evaluating number of characteristic features for each detected lesion. Numerous features evaluated may become a problem for machine learning algorithms, so optimal features are determined for potential nodule selection.

### 3.4. Classification

**Figure 5:**
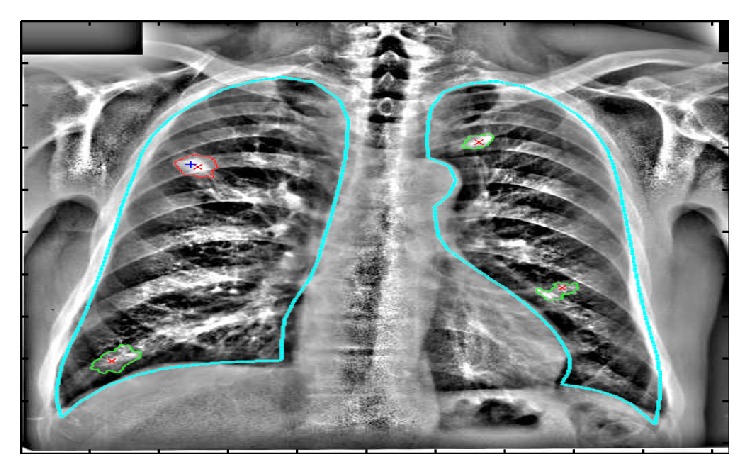
Potential nodule detection.

In this step out of detected regions, potential nodule candidates were selected using classifier. This needs a good classifier and a training set, which can enable the classifier to distinguish normal and abnormal regions. Correct classification is difficult because true candidate features space sometimes may exist in the false candidates and vice versa and there is no perfect classifier that exists for categorization. So developers always make trials with the existing methods.  Figure 5 shows the detected nodule candidates.

Giger ML, Doi K, and Mac Mahon H [22] followed pattern recognition technique for finding nodule candidate from set of suspected nodules. For each suspicious region degree of circularity and effective diameter is measured and potential candidate is detected with growth test and slope test. Growth test is based on degree of the circularity; if the degree of circularity is beyond certain cutoff level, then that suspected nodule is considered as nonnodule and is discarded. Then a slope test is performed to remove remaining false positives. Slope test is defined as the ratio change in effective diameter of the suspected nodule to the threshold level. Suspected nodule belonging to nonnodule will have highest slope rate. Based on slope rate nonnodules can be removed using predefined cutoff.

In most of the CAD schemes authors extracted features from suspected nodule candidate and using those features classifiers were trained and then used for discriminating predicted nodule regions as nodule and nonnodule regions. Different classifiers used in the literature are ANN, discriminant analysis, rule based classifiers, SVM classifier, fisher linear discriminant classifier, Bayes' classifier, etc. Wu Y, Doi K, Giger ML,[23] evaluated nine image features from each of the detected nodule candidates and extracted features used as input to the classifier for distinguishing true nodule from false positive detections. Automated classifiers used an artificial neural network, discriminant analysis, and a rule-based scheme. System eliminates 96% of false positive detections. Lo SC, Lou Sl, Lin JS, et al.[24] used artificial convolution neural network for classification. Penedo M.G, Carreira M.J, Mosquera A,* et al* [25] manipulated curvature peak features for each of the suspected nodule areas, using ANN false positive findings which were reduced. CAD works with 89%-96% sensitivity and 5-7 FPs/image. Coppini G, Diciotti S, Falchini M,* et.al*.[26] employed ANN with feed forward type for finding potential nodule and for reducing false findings, using nodule shape and back ground structures. Images from JSRT database were used for training and testing. Sensitivity of 60% to 75% and the number of false findings 4-10 per image were achieved with this system. Kenji Suzuki, Junji Shiraishi, Hiroyuki Abe, Heber MacMahon, and Kunio* et.al* [44] developed a classifier, which is Multi Massive Trained Artificial Neural Network, for reducing false positives from their previously proposed technique. Multi MTANN consists of several MTANNs in parallel. Each MTANN consists of three layers and is feed forward back propagation network. This trained Multi MTANN reduces 68.3% of false-positive findings with a reduction of one true positive result. Since ANN is used in this method, system requires more time for training and testing. System automation is not addressed in this method. Result shows multi MTANN designed can detect the nodules which are at the middle of the ROI; nodules existing in the corners of the ROI may not be detected. Method can discriminate nodules and nonnodules in an improved way. Zhenghao Shi, Minghua Zhao, Lifeng He, Yinghui Wang, Ming Zhangand Kenji Suzuki* et.al. *[28] used rule based classifier for removing elongated and small nodule regions from suspected nodules; these are considered as nonnodule regions. Later with MTSVM false positives are reduced. MTSVM consists of four nonlinear SVMs connected in parallel, with Gaussian radial basis function as kernel. To remove all major sources of false positives outputs of all the SVMs are combined with ANN. ANN consists of three layers: input, hidden, and output layers with identity, sigmoid, and linear functions. ANN is trained with back propagation training algorithm. Images from JSRT database are used for training and testing. Rule based classifier detects nodules with 85% sensitivity and 12 false positives per image. By using MTSVM false positives were reduced from 12 to 4. Author says here that system performance is further improved by incorporating anatomical features into the algorithm. A Gindi A., Rashed E., Sami M.*et.al*, [29] took help from radiologists for region of interest selection. ROIs selected are of different sizes because nodule size is not fixed. Discrete wavelet transform is used to extract features from each ROI. We use 4 different mother wavelet families: Daubechies, Haar, biorthogonal spline, and reverse–biorthogonal spline wavelets. For reducing the dimensionality of feature coefficients, the following steps were followed: (1) mean standard deviation, variance, covariance, and correlation coefficients were calculated for each level of decomposition; (2) a percentage of low frequency coefficients from each level of decomposition are selected; (3) selected features are arranged in descending order. In the final step using Euclidian distance classifier ROIs are classified into benign or malignant nodules. Results show biorthogonal and reverse biorthogonal wavelets yield better classification results compared to other wavelets used. Here experiments were conducted on real labeled data, those in hidden and visible regions. According to the results, proposed scheme is a very good classifier, and disadvantage here is that data to be tested and trained must be labeled by the radiologists; if radiologist was wrong in labeling the data, result may fail. Chen S., K. Suzuki and H. McMahon* et.al*,[30] evaluated 31 shape, gray level, surface, and gradient based features from each of the suspected nodule candidates. SVM classifier with Gaussian kernel was used for finding potential nodule out of all suspected nodule regions. Method can detect subtle and extremely subtle nodules with a sensitivity of 54.8% at an average of 5 false positives/image and obvious nodules were found with sensitivity of 91.1% and 2.6 false positives per image. CAD algorithm was trained with 300 images with nodules and 100 images without nodule. Images from JSRT database and University of Chicago were used for evaluation. Hardie R. C., S. K. Rogers, T. Wilson and A. Rogers,* et.al* [31] estimated 9 geometrical features, 18 intensity features, and 17 gradient features for all selected candidates. Fisher linear discriminant classifier was used for categorizing segmented regions as nodule or not a nodule. Images from JSRT database and Riverain Medical Center were employed for performance evaluation. Method can detect nodules with a sensitivity of 78.1% at an average of 4 false positives per image. Amal M. Al Gindi, Tawfik A. Attiatalla and Moustafa M. Sami* et.al.*[45] selected region of interests of size 128*∗*128 from the image with the help of 3 radiologists. For each ROI curvelet transform was applied and 10% of significant coefficients were selected; this was done for reducing the dimensionality of coefficients. Later with the help of two different classifiers, Euclidean distance and SVM classifiers nodule were classified into benign or malignant. Images used for testing and training belong to JSRT database. 50% of the images from the database were used for training, 30% of images from the database were used for testing. Result shows curvelet with SVM classifier shows good result compared to the curvelet with Euclidean distance classifier. Shiraishi, Q. Li, K. Suzuki, R. Engelmann, and K. Doi,* et.al *[32] extracted total of 71 image features from suspected nodule candidates employing three artificial neural networks to reduce number of false positive candidates; parameters of ANN like number of iterations, slope of sigmoid function, learning rate, and threshold values were evaluated automatically with boot strap technique for training cases. Average sensitivity obtained in detecting lung nodule was 70.1% with 5 F.P per image for test cases and 70.4% with 4.2 F.P image for training cases. A.Schilham, B. van Ginneken, and M. Loog,* et.al*.[33] applied blob detection method for finding suspicious nodule structures with the help of K nearest neighbor classification number false positives that were minimized. System can detect the potential nodule candidates with a sensitivity of 50.6% and about 2 false positives per image. J. Wei, Y.Hagihara, A.Shimizu, and H.Kobatake.*et.al.*[35] extracted four kinds of features like geometric features, contrast features, and first-order statistical and second-order statistical features from each of the suspected nodule candidates. Genetic algorithm for reducing dimensions of the features set was employed and the optimal features set was selected. True positive detection rate is 80% with 5.4 F.P per image. S.A. Patil, M. B. Kuchanur* et.al*.[36] extracted nodule regions from the image through thresholding, threshold value chosen using histogram. Then nodule is separated from the radiograph with region growing technique in case of non-small cell lung cancer images; in case of small cell lung cancer region labeling was employed. For reducing artifacts present in the image several morphological operations were applied on the image. From the segmented tumor features like area, perimeter, diameter, and irregularity index have been estimated. In addition to this first-order statistic texture features such as average gray level, standard deviation, smoothness, third moment, uniformity, and entropy and second-order texture features like contrast, correlation, energy, and homogeneity are manipulated using gray Level cooccurrence matrix. For TB analysis lung region is divided into 4 equal parts; for each region separately first-order and second-order statistical texture features were manipulated. Using these estimated features with the help of ANN type of abnormalities is identified. There were 83% classification accuracy results with the training data. H. Khanan Nehemiah* et.al.* [46] developed two subsystems: nodule detection subsystem and nodule validation subsystem. Input to NDS system is chest radiograph of size 512*∗*512. NDS was further divided into 3 subsystems: image denoising engine, segmentation engine, and nodule recognition engine. Output of NDS is suspected nodule regions, given to nodule validation subsystem, where ANN is used to classify the nodule into cancerous or noncancerous. Based on classification of an identifier attached to each PA chest radiograph, identifier represents characteristics of the nodule. For these 100 images, of the algorithm, used for training, neural network can classify 38 images correctly as true positives, 4 images are classified as true negatives, and 8 images are classified as false positives.

Carlos S. Pereira, Luís A. Alexandre, Ana Maria Mendonça and Aurélio Campilho A. Campilho and M. Kamel (Eds.)* et. al *[47] followed multiclassifier approach to classify the regions in chest radiographic images as nodule or nonnodule regions. Classification is done here in two steps, primarily using multiscale and multiorientation filter bank; rotation invariant features are calculated; later by using different classifiers like multilayer perceptrons regions they are classified. The assumption that author followed here is that frequency spectrum of a textured image is different from the other distinct textures. Bank of Gabor filters was used for extracting image features; each bank contains certain number of Gabor filters. Multiple classifiers based on different multilayer perceptrons are used and each classifier was fed with a different set of features. Outputs from these classifiers were combined to generate final classification decision. In this work images from JSRT database have been used for testing and training. Reportedly 72% of detection rate was achieved. Preetha.J, G. Jayandhi et.al. [48] proposed a method for rib suppression; nodule was detected from rib suppressed image with Bayes' classifier. By using active shape model nodule regions were segmented. For each suspicious region different feature like circularity index, mean intensity, average contrast, smoothness, skewness, and entropy were manipulated. With the help of these features using the above classifier segmented nodule regions are classified into nodule or nonnodule regions. Zakaria Suliman Zubi, Rema Asheibani Saad [38] extracted possible nodule regions from the visible lung region by applying thresholding and a series of morphological operations. Only 3 features are extracted from each suspected nodule, area, perimeter, and shape. Artificial neural network is used as classifier, trained using back propagation training algorithm. 60 X-ray chest images from multimedia database were used for testing and training. 70% of images were used for training and 15% for testing. Results show method can categorize benign nodules with 95% accuracy and malignant nodules with 85% accuracy. Hamada R. H. Al-Absi and Brahim Belhaouari Samir* et.al *[2] selected ROIs of size 128*∗*128 from the original image. Selected regions are preprocessed using Laplacian of Gaussian filter. Later from each ROI, using wavelet transform with db1 wavelet, coefficients are extracted. Wavelet decomposition is done up to 6 levels. Similarly using curvelet transform up to 7 scale coefficients are obtained. Required coefficients from each transform are selected by evaluating statistical energy and statistical metric. Selection of coefficients using statistical metric is based on threshold. Finally KNN classifier is used to classify the regions as nodule or nonnodule and also as malignant or benign. Result shows wavelet transform distinguishes the suspected nodule as a nodule or nonnodule with an accuracy of .9915 using db1 wavelet, and it differentiates the nodule as malignant or benign with an accuracy of .9481, whereas by applying curvelet accuracy of .7692 is obtained, while classifying suspected nodule as nodule or nonnodule. And an accuracy of .9091 is obtained while distinguishing nodule as malignant or benign. G. Orbán1, Á. Horváth1 and G. Horváth1* et.al* [39] applied nodule detection algorithm on bone eliminated images. Constrained sliding band filter is used to enhance the possible nodule regions. Regions which are having high CSBF value are considered as suspected nodule regions. For each subregion features such as contrast, angular second movement, and entropy related measurements and average fraction under minimum filter output were evaluated. These features are used as input for the SVM classifier to differentiate subregion as nodule or nonnodule. Result shows performance of the classifier is in equivalence with the existing algorithms, with sensitivity of 61% with 2.5 false positives per image. Paola Campadelli and Elena Casiraghi* et.al* [49] proposed multiscale algorithm for detecting possible lung nodules. 12 features are obtained for each suspicious region; those are features based on shape, gray level distribution, position, etc. ANN is trained with the 12 features, classifying each suspicious region as nodule or nonnodule. The best result obtained with this method was false positives reduced from 32000 to 11000 after testing 247 images. Hiroyuki Yoshida, Bilgin Keserci, and Kunio Doi* et.al* [50] divided image into subregions of size 64*∗*64. Region of interest consists of 84 true positives and 694 false positives. For nodule candidate in ROI wavelet snake algorithm was applied to fit into boundaries of the candidate. The degree of overlap between multiscale edge obtained by applying spline wavelet to ROI and fitted snake is a measure for distinguishing nodule and false positives. ANN was trained with this measure and morphological features to find potential nodule candidate and to reduce false positives. Performance of the system was analyzed using receiver operating characteristics. Wavelet snake combined with morphological features gives good results compared with morphological features alone. G. Coppini, S. Diciotti, M. Falchini, N. Villari, and G. Valli* et.al* [27] biologically inspired filters like Laplacian of Gaussian filter and Gabor filter which were applied to improve the image features of the image. ANN is used to classify the nodules using shape features. Images tested here were from JSRT database. Algorithm produces sensitivity of 60 to 75% with 4 to 10 false positives per image. M. Aoyama, Q. Li, S. Katsuragawa, H. MacMahon, and K.Doi* et.al* [51] developed an algorithm to discriminate nodule identified with the help of radiologists into benign and malignant. Recognized nodule locations were segmented by employing difference imaging technique and automatic analysis of contour line distribution. 75 features were evaluated for each nodule; nodule regions were classified using ANN and LDA classifier. Linear discriminant analysis shows good result: Katsumi Nakamura Hiroyuki Yoshida, Roger Engelmann, Heber MacMahon Shigehiko Katsuragawa, Takayuki IshidaKazuto Ashizawa, and Kunio Doi,*et.al* [52].

Authors presented a method to classify nodule in posterior and anterior chest radiograph into benign and malignant. Eight subjective features like nodule size, shape, marginal irregularity, speculation, border definition, lobulation, density, and homogeneity are evaluated with help of radiologists. Computerized methods were used to find features similar to features found by radiologists. ANN was trained with subjective or objective features to classify nodule as benign and malignant. ANN shows better results with objective features compared with subjective features. Paola Campadelli*, Member, IEEE*, Elena Casiraghi, and Diana Artioli* et.al* [34] applied multiscale method to enhance the appearance of the nodule. SVM classifier was employed for detecting potential nodule candidate and to reduce false positives. Gaussian and polynomial SVMs were trained with different parameters; good SVM model gives sensitivity of .71 and 1.5 false positive per image; as sensitivity increases to .92 false positives per image increase to 7 to 8 per image. K.A.G. Udeshani, R.G.N. Meegama and T.G.I. Fernando* et.al.*[40] applied otsu method on an image for converting an image into binary mage. Possible nodule regions were estimated by means of circularity index of each connected component. First-order and second-order statistical features were evaluated for each suspected nodule region. Statistical features and pixel intensity values of the region were used for training and testing an ANN, to find whether detected region was nodule or not a nodule. Method detects the nodules with an accuracy of 96%. Nitin S. Lingayat and Manoj R. Tarambal,* et.al* [41] proposed an algorithm in which nodule regions were identified by thresholding, edge detection, and labeling. For each identified region area, perimeter, irregularity index, equivalent diameter, convex area, solidity, and statistical features were evaluated. Suspicious regions were classified as malignant or benign with the help features evaluated. Author concluded nodule as benign when tumor area, and perimeter was larger and irregularity index is higher compared to malignant. Entropy is higher for malignant tumor. M. S. Ahmad, M. Shahid Naweed and M. Nisa* et.al*.[53] evaluated different parameters from each suspicious nodule candidate using image histogram, cooccurrence matrix, and wavelet analysis. Best features selection was done with principal component analysis and linear discriminant analysis. Final classification was done with ANN.

Kim Le* et.al*.[42]developed a method in which for every lung pixel fixed size window is applied; average and maximum gray level values of the pixels inside the window were calculated. The value between average and maximum gray level values is selected as threshold value. Pixels which are having gray level values greater than threshold were marked. Number of pixels greater than threshold is counted; if the count is greater than predefined threshold that set of pixels is considered as suspected nodule. Conclusions were made that algorithm works well, for finding early nodules from lungs, TB, and congestive heart failure.

Most of the CAD schemes in the literature are suffering from false positives. These may be due to rib and rib crossings, rib and vessel crossings, and end-on vessels. For reducing the effect of these on CAD algorithm, different techniques were proposed.

Jyh-Shyan Lin, Akira Hasegawa, Matthew T. Freedman, and Seong K. Mun* et.al*.[54] developed an algorithm which differentiates an end on vessel from nodule, thereby decreasing the false positives due to end vessels. End-on vessel appears bright in the image compared to nodule of same size. Images used for testing are from Georgetown University Medical Center. To reduce the image processing complexity images were resized to 512*∗*625. Images blocks of size 32*∗*32 are extracted manually from cancerous and noncancerous PA chest radiographic images. Image blocks are taken by excluding the helium area. Since image blocks are taken from different parts of the image, they are of different brightness levels; background trend correction technique is employed to correct the brightness levels. Convolution neural network is used here for differentiating nodule and end-on vessel. CNN is trained and tested with pixel values of the image patches. CNN was trained using stochastic gradient procedure. 40 patches containing nodules and 53 patches containing end-on vessels are used for training. 66 nodule patches and 46 end-on vessels are used for testing. Desired output value is 1 or 0 for nodule or end-on vessel. Report shows that performance of this algorithm is more accurate compared to the radiologists observations in distinguishing nodule from end-on vessels. Elaheh Soleymanpour, Hamid Reza Pourreza, Emad Ansaripour, Mehri Sadooghi Yazdi* et.al* [1] applied spatial Gabor filter on radiographic image for suppressing the appearance of ribs in the image and to enhance the conspicuity of the nodule regions. Bilal Ahmed* et.al* [55] presented rib suppression method, which uses fast independent component analysis algorithm for removing ribs and clavicles from the image. Suppressing ribs enhances the remaining parts of the image. Áron Horváth* et.al* [56] followed dynamic programming approach for separating ribs and clavicles from chest radiographic image. Segmented ribs and clavicles shadows were used for removing the same from the image using difference imaging technique. Hybrid lesion detector was designed based on gradient convergence, contrast, and intensity statistics used for finding possible nodule regions. M.Loog, B.Van Ginneken[57] presented a scheme to suppress the bony structures from posterior and anterior chest radiographs. Technique was based on k-nearest neighbor regression. System was trained initially with dual energy radiographs, using dual energy faking method. Technique was based on k-nearest neighbor regression. System was trained initially with dual energy radiographs

Sheng Chen and Kenji Suzuki* et.al* [58] developed Massive Trained Artificial Neural Network to eliminate posterior and anterior chest radiographs. MTANN was developed as multilayer ANN regression model which operates on each pixel data of the input image. Input image is divided into overlapping subregions using active shape model technique. Each MTANN in a set of MTANNs is trained with one of the subregions of the input image; teaching image is a bone image which contains enhanced ribs. Here total of 9 images are used for training 8 images containing nodule; one image does not contain any nodule. While testing the image, image is separated into small overlapping segments (number of segments chosen in this paper is 8); each segment is used as input for each MTANN. Outputs of all eight MTANNs are combined and are smoothed by a Gaussian filter. Resultant bone image is called virtually dual energy image. Further noise in the image is minimized, while preserving edges. Obtained VDE bone image is subtracted from the original image to get image without ribs while preserving the conspicuity of the nodule and vessels in the image. This method is tested on 110 radiographic images.

## 4. Conclusions

PA chest radiography is the cheapest method of diagnosing any abnormalities from chest and is also available in all diagnostic centers and requires less radiation dose compared to the remaining chest imaging techniques. So this becomes the most frequently used technique for examining the chest abnormalities. Disadvantage of PA chest radiography is complex anatomical structures, due to the fact that even experienced radiologists could not detect the nodules correctly from radiographic image. Rib crossings, end-on vessels, vessel and vessel crossings, and rib and vessel crossings mislead radiologists while examining radiographs. So to help radiologists in detecting tumor from images, for decades numerous computerized algorithms were presented in the literature. Some of those algorithms were discussed in this paper. We developed different methods [59, 60] for detecting tumor from chest radiographs. In one of our proposed algorithms, Circular Hough Transform was used for finding nodule regions, wherein shape of the nodule regions present in the image is presumed as circular. And in another proposed method entire radiograph is divided into subsections of fixed size. Restricted Boltzmann machine and SVM classifier were used to classify subsections into nodule and nonnodule regions.

Though great research has been done in the development of computerized systems, all the proposed methods suffer from miss detection of nodule from image and higher number of false positives per image. This is due to appearance of the nodule in different size and also with different intensity and complex anatomical structure present in the image. For reducing the effect of complex anatomical structures on the proposed CAD systems, the authors followed different rib suppression technique but those methods were also not showing accurate results. So the problem is still open and future research must focus on reducing false positives and false negatives created by computerized analysis.

## Data Availability

The data used to support the findings of this study are included within the article.
